# Extrauterine Growth Restriction in Very Low Birth Weight Infants: Concordance Between Fenton 2013 and INTERGROWTH-21^st^ Growth Charts

**DOI:** 10.3389/fped.2021.690788

**Published:** 2021-06-21

**Authors:** Lara González-García, Enrique García-López, Belén Fernández-Colomer, Laura Mantecón-Fernández, Sonia Lareu-Vidal, Marta Suárez-Rodríguez, Rosa Patricia Arias-Llorente, Gonzalo Solís-Sánchez

**Affiliations:** Central University Hospital of Asturias, Oviedo, Spain

**Keywords:** newborn, extrauterine growth restriction, intrauterine growth restriction, very low birth weight, nutrition

## Abstract

Postnatal growth restriction has high prevalence in very low birth weight (VLBW) preterm neonates, and this could affect their long-term prognosis. Nowadays, there is no consensus on how to monitor growth in these neonates.

**Objective:** This study aimed to compare prevalence of intra- and extrauterine growth restriction (IUGR and EUGR) in a sample of VLBW infants according to the Fenton 2013 charts and INTERGROWTH-21st (IW-21) standards and to analyze concordance between both in the different EUGR definitions criteria (cross-sectional, dynamic, and true).

**Patients and Methods:** An observational retrospective study of 635 VLBW preterm was performed. The study was carried out in Central University Hospital of Asturias. Body measurements (weight, length, and head circumference) were collected at birth and at hospital discharge and expressed in z-scores for the two references (Fenton 2010 and IW-21). Kappa concordance was calculated.

**Results:** Kappa concordance between Fenton and IW-21 was 0.887 for IUGR and 0.580 for static EUGR. Prevalence was higher according to Fenton in IUGR (36.5 vs. 35.1%), in static EUGR (73.8 vs. 59.3%), and in dynamic EUGR (44.3 vs. 29.3%). Despite observing low prevalence of EUGR when IW-21 was used to define EUGR, a statistical association between neonatal morbidity and diagnosis of EUGR was observed.

**Conclusion:** The Fenton and IW-21 concordance for IUGR is good. IW-21 is more restrictive than Fenton in EUGR. Patients diagnosed by IW-21 as EUGR are more likely to have neonatal morbidity, especially if we use EUGR dynamic definition. In our study, we cannot conclude that one graph is better than the other.

## Introduction

Preterm infants are at risk of extrauterine growth restriction (EUGR) as a consequence of their own intrauterine growth restriction (IUGR), immaturity, and related morbidities, usually associated with food intolerance, inadequate nutrition, and elevated metabolic needs during their hospital admission (1, 2). These changes in growth could have short- and long-term consequences such as growth failure, cardiovascular risk, and developmental disabilities (3–6).

Today, there is no international consensus regarding how to monitor growth of premature infants, especially in those who are very low birth weight (VLBW) infants (7, 8), which in turn are the group of premature babies with the highest risk of growth disturbance. Doubts arise in two directions: what graphs or standards to use as normality reference and what criteria to use to classify IUGR and EUGR.

Since 1977, the American Academy of Pediatrics (AAP) has recommended the use of fetal growth charts during pregnancy to monitor postnatal growth. However, it is not usual for a premature infant to present the same postnatal growth pattern once born. With these references, a high percentage of VLBW infants will be classified as EUGR at discharge (9, 10). VLBW infants usually often fail to gain weight as expected based on intrauterine growth charts (7, 11).

By now, Fenton growth charts have been the most used references to monitor postnatal growth. They were made with somatometric data obtained at birth from fetus according to gestational age (GA) and sex from almost 4 million births from different countries, and they were updated back in 2013. The Fenton charts continue with the World Health Organization (WHO) growth charts at 50 weeks postmenstrual age (PMA). These charts have variability in the measurement methods and do not take into account the physiological loss of weight that occurs after delivery (12).

In recent years, a paradigm shift has emerged. It is preferred to use growth standards of healthy preterm infants than graphics based on cross-sectional somatometric data from fetus at birth (8). Based on these recommendations, the INTERGROWTH-21st Proyect (IW-21) was made prospectively with postnatal growth standards. IW-21 charts include patients from eight countries and overlap WHO growth charts at 64 PMA and are universally applicable (8, 13). In IW-21 growth standards, whose data were prospectively collected between 2009 and 2014, low-risk healthy women who conceived spontaneously with a reliable estimate GA from first trimester without IUGR were eligible to participate. Standardized anthropometric measurements were made in preterm births from this cohort (13).

Although IW-21 charts seem to be better than Fenton, their use is not widespread in daily practice. Besides, the growth chart we use (Fenton vs. IW-21) will influence the prevalence of IUGR and EUGR because this varies widely according to the standards used to monitor postnatal growth (14, 15).

On the other hand, there is no consensus in how to define EUGR. It can be defined in two ways: transversal (cross-sectional) or longitudinal (dynamical). The cross-sectional definition uses a specific time (typically at time of discharge or at 36 weeks PMA) and includes those patients having a weight below the 10th percentile. The longitudinal (dynamical) definition includes those patients with a weight loss of more than 1 or 2 standard deviation (SD) from birth to discharge or at 36 weeks PMA. Some studies point out a better prognostic utility when dynamic definition is used (14). Recently, a new concept of “true EUGR” has started to be discussed: non-IUGR patients at birth are EUGR at 36 weeks or discharge. This new “true EUGR” avoids EUGR patients with IUGR at birth who probably does not have a growth problem of postnatal origin, maybe as a result of a continuation of impaired growth that began at fetal time (16).

Given the high incidence of EUGR during neonatal intensive care unit (NICU) stay in VLBW infants and its possible effect in long-term growth (17) and neurological development (5, 6), it is very important to define which growth chart must be used and how to better define EUGR to monitor the postnatal growth. For this reason, the objective of our study was to compare IUGR and EUGR (static, dynamic, and true) prevalence according to the Fenton and IW-21 standards, looking for concordance between both and analyzing neonatal factors associated with these classifications.

## Materials and Methods

**Figure 1:**
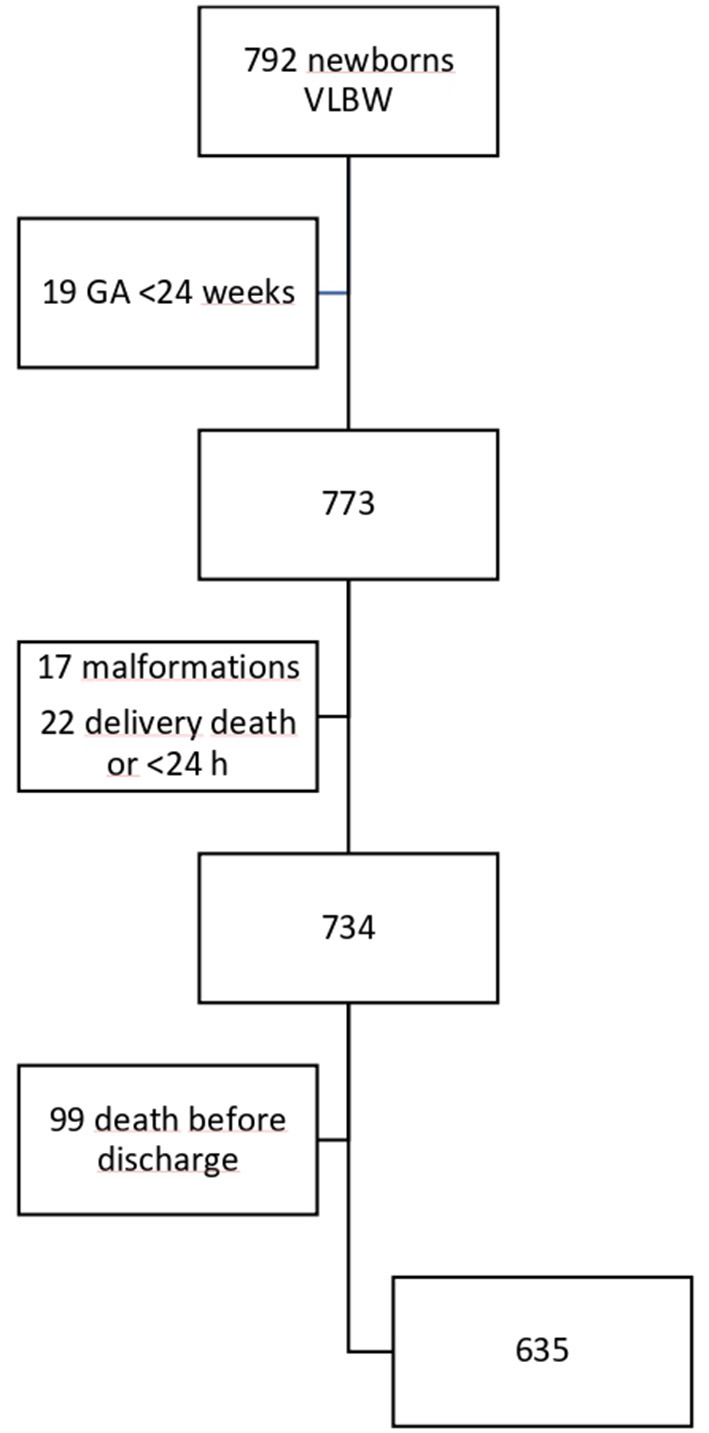
Flowchart of the sample according to inclusion criteria and study population. VLBW, Very low birth weight.

A clinical retrospective study was designed. A total of 792 VLBW preterm neonates who weighed <1,500 g at birth were eligible to participate in the study during a period of 16 years, from January 2002 to December 2017. The study was carried out in the Neonatology Unit of the Central University Hospital of Asturias (Oviedo, Spain), a third-level hospital that is reference for a population of 1 million inhabitants and with about 5,000 deliveries a year. From the initial population, 635 patients were finally studied (Figure 1 shows the flowchart of the included patients).

All patients were included at birth in a perinatal morbidity database (SEN1500) after an informed consent was given their parents or legal guardian (18). Exclusion criteria were as follows: GA <24 weeks, death before hospital discharge, major congenital malformations, chromosomopathies, and congenital embryopathies with growth impairment (such as congenital infection by cytomegalovirus).

The study has been carried out in accordance with The Code of Ethics of the World Medical Association (Declaration of Helsinki) and was approved by the Research Ethics Committee of Principado de Asturias (CEIm PA, SPAIN). The recommendations of the Spanish Society of Neonatology were followed at all times regarding the early introduction of trophic and parenteral nutrition. It should be noted that the study took place in a period of 16 years, so nutrition recommendations have been changing, adapting to current international recommendations. Parenteral nutrition and trophic enteral nutrition were introduced in the first 24 h of life, fortifying breastfeeding when an enteral feeding volume reached 100 ml/kg/day.

Weight (W), length (L), and head circumference (HC) were expressed in mean and SDs (z-scores) using the Fenton 2013 and IW-21 references at birth, at 28 days, and at hospital discharge. In one male patient, the z-score for Fenton at discharge could not be calculated because he was discharged at 60 weeks GA (z-score unavailable). Neonates were classified as IUGR if their birth weight was below the 10th percentile. Body mass index (BMI) was calculated using the weight and length data using the following formula: (g/cm^2^) ^*^ 10. Subsequently, the z-score BMI was calculated using the Olsen references (19). Values were expressed as mean and SD.

EUGR was defined in a transverse-static way (weight at discharge below the 10th percentile using the Fenton and IW-21 references) and longitudinally dynamically (decrease >1 SD between birth and hospital discharge using Fenton and IW-21). “True EUGR” was defined as the EUGR (static or dynamic criteria) in non-IUGR patients.

### Statistical Analysis

Data were analyzed using IBM SPSS statistical software, version 22.0 (IBM®). Quantitative variables were expressed as mean with SD, and qualitative variables as absolute number and percentage. Chi-square test was used for the comparison of qualitative variables, while for the quantitative variables, parametric tests (Student's *t*-test) were used when the sample followed a normal distribution. Kappa coefficient was used to see the concordance between the IW-21 and Fenton references.

Receiver operating characteristic (ROC) curves were used to compare the discriminatory power of the decrease in z-score in the first 28 days of life in EUGR prediction. Area under the curve (AUC) was calculated for each decrease in z-score to diagnose the outcome variable (EUGR in its different definitions) in both graphs.

Multivariate logistic regression (enter method) analysis for dynamic and static “true EUGR” in weight was conducted in each growth chart to determine factors influencing “true EUGR.” The significance level adopted was 5%.

## Results

Mean GA was 30.2 ± 2.5 weeks. Mean W, L, and HC at birth were 1,173 ± 239 g, 38.1 ± 3 cm, and 26.4 ± 2.1 cm, respectively. Two hundred eight cases (32.8%) came from multiple births and 107 (16.9%) from *in vitro* fecundation gestation. A proportion of 57.8% (367) of the mothers completed a full dose of prenatal corticosteroids, and 72.9% (463) delivered by cesarean section. Morbidities during the neonatal period can be seen in Table 1.

**Table 1:** Characteristics of the study population.

		**Value**
Gestational age	At birth (weeks)	30.2 ± 2.5
	<30 weeks at birth	284 (44.7)
	PMA at discharge (weeks)	38.7 ± 2.7
Somatometry at birth	Weight (g)	1,173 ± 239
	Length (cm)	38.1 ± 3
	Head circumference (cm)	26.4 ± 2.1
	BMI (g/cm^2^)	7.97 ± 0.91
Somatometry at discharge	Weight (g)	2,416.2 ± 307.5
	Length (cm)	45.6 ± 2.1
	Head circumference (cm)	33.2 ± 1.5
	BMI (g/cm^2^)	11.56 ± 1.09
Perinatal data	Male gender	306 (48.2)
	Prenatal corticosteroids (complete)	367 (57.8)
	Multiple gestation	208 (32.8)
	Cesarean section	463 (72.9)
	Apgar score 5 min <5	20 (3.1)
	Crib 1 score median (RIQ)	1 (1, 2)
	Intubation resuscitation	224 (35.4)
Neonatal pathology	RDS	307 (48.3)
	MV	345 (54.3)
	Pneumothorax	20 (3.1)
	Early-onset sepsis	24 (3.8)
	Late-onset sepsis	201 (31.7)
	Anemia (transfusion)	176 (33.6)
	NEC	21 (3.3)
	PDA	144 (22.7)
	Hypotension (inotropic support)	45 (7.1)
	Acute kidney injury	14 (2.2)
	Parenteral nutrition at 28 days of life	54 (8.5)
	ROP ≥ 2 stage	62 (9.8)
	BPD	125 (19.7)
	PVL	58 (9.1)
	HIV grade 3–4	27 (4.3)

*Values are expressed as number (%) or mean ± standard deviation*.

*BMI, body mass index; CRIB, Clinical Risk Index for Babies; RDS, respiratory distress syndrome; MV, mechanical ventilation; NEC, necrotizing enterocolitis; PDA, patent ductus arteriosus; ROP, retinopathy of prematurity; BPD, bronchopulmonary dysplasia; PMA, postmenstrual age; PVL, periventricular leukomalacia; HIV, intraventricular hemorrhage*.

The proportion of children identified as IUGR using the 10th percentile of the IW-21 references for W, L, and HC was 35.1, 29.8, and 34.8%, respectively, and was 36.5, 26.8, and 34.8%, respectively, using Fenton. Kappa concordance between both was 0.887 for W, 0.856 for L, and 0.806 for HC (Table 2).

**Table 2:** IUGR comparison using Fenton and IW-21 (weight, length, and head circumference).

			**INTERGROWTH-21**^****st****^			**Kappa**
			**IUGR**	**Non-IUGR**	**Total**	
Fenton 2013	Weight	IUGR	211 (33.2)	21 (3.3)	232 (36.5)	0.887
		Non-IUGR	12 (1.9)	391 (61.3)	403 (63.5)	
		Total	223 (35.1)	412 (64.9)	635	
	Length	IUGR	161 (25.4)	9 (1.4)	170 (26.8)	0.856
		Non-IUGR	28 (4.4)	436 (68.8)	464 (73.2)	
		Total	189 (29.8)	445 (70.2)	634	
	HC	IUGR	193 (30.4)	28 (4.4)	221 (34.8)	0.806
		Non-IUGR	28 (4.4)	386 (60.8)	414 (65.2)	
		Total	221 (34.8)	414 (65.2)	635	

*Values are expressed as number (%)*.

*IUGR, intrauterine growth restriction; HC, head circumference*.

Patients identified by Fenton and IW-21 (both) as IUGR compared with the rest of the patients (non-IUGR in both) had a significantly higher GA and presented fewer complications associated with prematurity [lower risk of respiratory distress syndrome (RDS), intubation in resuscitation at birth, bronchopulmonary dysplasia (BPD), need for parenteral nutrition at 28 days, intraventricular hemorrhage (IVH), and sepsis] (Table 3). Comparing the patients who were only identified by Fenton with those who were only identified by IW-21, there were no significant differences except a predominance of male and a significantly lower W at birth in IW-21.

**Table 3:** Comparison of neonatal morbidity in IUGR and non-IUGR patients for both charts.

	**IUGR for both charts**	**Non-IUGR in both charts**	***p***
	**(*n* = 211)**	**(*n* = 424)**	
Male sex	103 (48.8)	203 (47.9)	NS
Gestational age (weeks)	32.3 ± 2.2	29.1 ± 2	<0.0001
Birth weight (g)	1,139 ± 266	1,189 ± 223	0.02
Weight 28 days (g)	1,569 ± 434	1,444 ± 363	<0.0001
Weight at discharge (g)	2,312 ± 168	2,468 ± 345	<0.0001
Length of stay (days)	52.7 ± 23.5	63.63 ± 27.2	<0.0001
Apgar score 5 min <5	6 (2.8)	14 (3.3)	NS
Intubation resuscitation	50 (23.7)	175 (41.4)	<0.0001
RDS	49 (7.7)	258 (40.6)	<0.0001
MV	63 (29.9)	283 (66.7)	<0.0001
Pneumothorax	2 (0.9)	18 (4.2)	0.025
Early-onset sepsis	2 (0.9)	22 (5.2)	0.008
Late-onset sepsis	51 (24.2)	150 (35.4)	0.004
Anemia (transfusion)	46 (26.1)	130 (37.4)	0.01
NEC	5 (2.4)	16 (3.8)	NS
PDA	16 (7.6)	128 (30.2)	<0.0001
Hypotension (inotropic use)	10 (4.7)	37 (8.7)	NS
AKI	5 (2.8)	9 (2.6)	NS
Parenteral nutrition at 28 days	12 (5.7)	42 (10)	0.001
ROP ≥ stage 2	16 (9.5)	46 (11.5)	NS
BPD	21 (10.1)	104 (24.5)	<0.0001
PVL	17 (8.1)	41 (9.7)	NS
HIV grade 3–4	2 (0.9)	25 (5.9)	0.004

*Values are expressed as number (%) or mean ± standard deviation*.

*IUGR, intrauterine growth restriction; RDS, respiratory distress syndrome; MV, mechanical ventilation; NEC, necrotizing enterocolitis; PDA, patent ductus arteriosus; AKI, acute kidney injure; ROP, retinopathy of prematurity; BPD, bronchopulmonary dysplasia; PVL, periventricular leukomalacia; HIV, intraventricular hemorrhage*.

If we focus on IUGR, IUGR patients met the static EUGR criteria in 97% of cases using the Fenton graphs and in 87.9% using IW-21. However, IUGR was a protective factor for the development of dynamic EUGR, occurring only in 29% using Fenton and in 10.8% using IW-21.

### Static Extrauterine Growth Restriction (<10th Percentile at Hospital Discharge)

With a cross-sectional cutoff point, the proportion of infants identified as EUGR according to Fenton and IW-21 with respect to W, L, and HC was 73.7 and 53.9%, 63.5 and 57.6%, and 23.5 and 25.9%, respectively. Furthermore, 20% (127 children) identified by Fenton as EUGR had no IW-21 EUGR. Kappa concordance between both classifications was 0.58 in W, 0.803 in L, and 0.852 in HC.

A third of all static EUGR patients (30.9% in IW-21 vs. 35.5% in Fenton) had previous history of IUGR.

Analyzing the subgroup of patients with birth weight <1,000 g (*N* = 161), we observed that only 23.6% (38) achieved a discharge weight above the 10th percentile using IW 21 vs. 13% (20) using Fenton, maintaining good concordance between both classifications (Kappa = 0.613) (Table 4).

**Table 4:** Static EUGR in weight (<10th percentile at hospital discharge) in <1,000 g.

		**INTERGROWTH-21st**			**Kappa**
**Fenton 2013**		**EUGR**	**Non-EUGR**	**Total**	
	EUGR	122 (75.8)	18 (11.2)	140 (87)	0.613
	Non-EUGR	1 (0.6)	20 (12.4)	21 (13)	
	Total	123 (76.4)	38 (23.6)	161	

*Value is expressed as number (%)*.

*EUGR, extrauterine growth restriction*.

### Dynamic Extrauterine Growth Restriction (Decrease > −1 SD at Hospital Discharge)

Dynamic EUGR (decrease in more than 1 SD between birth and hospital discharge) according to Fenton and IW-21 for W, L, and HC was 44.3 and 29.3%, 58.3 and 43.8%, and 13.7 and 12.6%, respectively. We observed that 15.3% of children diagnosed as dynamic EUGR in the Fenton charts for W did not have dynamic EUGR according to IW-21. Kappa concordance in dynamic EUGR diagnosis was 0.672 for W, 0.619 for L, and 0.704 for HC.

Table 5 shows the prevalence of IUGR, EUGR (static and dynamic), and “true EUGR” (static and dynamic), expressing Kappa concordance.

**Table 5:** Prevalence of IUGR, EUGR (static and dynamic), and “true EUGR” according to Fenton 2013 and INTERGROWTH-21st and Kappa concordance between both classifications.

	**IUGR**	**EUGR**			
		**Static**	**Dynamic**	True^*^ static	True^*^ dynamic
**Weight**					
Fenton	36.5	73.8	44.3	59.2	52.8
IW-21	35.1	53.9	29.3	34.9	41
Kappa	0.887	0.58	0.672	0.539	0.746
**Length**					
Fenton	26.8	63.6	58.6	47.9	66.2
IW-21	29.8	57.6	43.8	42.8	45.6
Kappa	0.856	0.803	0.619	0.804	0.571
**Head circumference**					
Fenton	34.8	23.5	13.7	14.4	19.2
IW-21	34.8	25.9	12.6	15.9	15.4
Kappa	0.806	0.852	0.704	0.86	0.723

*IUGR, intrauterine growth restriction; EUGR, Extrauterine growth restriction; IW-21, INTERGROWTH-21^st^; Fenton, Fenton 2013*.

* *True EUGR excludes IUGR (denominator IW-21 and Fenton are n = 412 and n = 402, respectively)*.

### True Extrauterine Growth Restriction

True static EUGR (EUGR prevalence in non-IUGR VLBW) was 35.7% using IW-21 standards and 60.4% using the Fenton curves.

Patients who experienced static true EUGR according to IW-21, in comparison with non-true EUGR, were more frequently male; had lower GA and lower birth weight; had longer hospitalization stays; and frequently suffered from more retinopathy of prematurity (ROP) ≥stage 2, necrotizing enterocolitis (NEC), mechanical ventilation, BPD, late-onset sepsis, hypotension, anemia requiring transfusion, acute kidney injury (AKI), patent ductus arteriosus (PDA), and RDS; and had more parenteral use at 28 days of life; and their BMI was significantly lower. Patients who experienced static true EUGR using Fenton had experienced similar comorbidities, although no association was seen with hypotension, AKI, and parenteral nutrition use at 28 days of life (Table 6).

**Table 6:** Static and dynamic “true^*^ EUGR” compared with “true^*^ non-EUGR” and their comorbidities for INTERGROWTH-21st and Fenton 2013.

	**Static true EUGR**		**Dynamic true EUGR**	
	**IW-21**	**Fenton**	**IW-21**	**Fenton**
	***N* = 147 (35.7)**	***N* = 243 (60.4)**	***N* = 163 (39.6)**	***N* = 212 (52.7)**
Male sex	86 (58.5)	129 (53.1)	97 (59.5)	114 (53.8)
Gestational age (weeks)	28.76 ± 2.20	29.08 ± 2.1	28.21 ± 1.99	28.38 ± 1.96
Birth weight (g)	1,104 ± 236	1,144 ± 232	1,118.6 ± 230.3	1,121.46 ± 225.19
z-score BMI at birth	−0.56 ± 0.84	−0.42 ± 1.02	−0.08 ± 0.9	−0.15 ± 0.94
z-score BMI at discharge	−1.18 ± 0.87	−1.06 ± 0.69	−1.1 ± 0.76	−1.03 ± 0.75
Length of stay (days)	78.7 ± 34.3	69.3 ± 28.9	79.37 ± 31.59	74.46 ± 28.03
Intubation resuscitation	80 (54.4)	117 (48.1)	103 (63.2)	123 (58)
RDS	106 (72.1)	171 (70.4)	132 (81)	163 (76.9)
MV	117 (79.6)	178 (73.3)	144 (88.3)	177 (83.5)
Early-onset sepsis	7 (4.8)	14 (5.8)	10 (6.1)	11 (5.2)
Late-onset sepsis	71 (48.3)	102 (42)	74 (45.5)	91 (42.9)
Anemia (transfusion)	72 (59.5)	92 (45.8)	79 (59.8)	95 (54.3)
NEC	10 (6.8)	14 (5.8)	13 (8)	13 (6.1)
PDA	59 (40.1)	84 (34.6)	75 (46)	82 (38.7)
Hypotension (inotropic support)	19 (12.9)	24 (9.9)	23 (14.1)	22 (10.4)
Acute kidney injury	8 (6.7)	6 (3)	6 (4.5)	5 (2.9)
Parenteral nutrition at 28 days	24 (16.4)	30 (12.4)	33 (20.4)	36 (17.1)
ROP ≥ stage 2	30 (20.8)	34 (14.6)	32 (19.8)	39 (18.7)
BPD	53 (36.1)	75 (30.9)	66 (40.5)	78 (36.8)
PVL	16 (10.9)	23 (9.5)	22 (13.5)	25 (11.8)
HIV grade 3–4	12 (8.2)	14 (5.8)	16 (9.8)	17 (8)

*Values are expressed as number (%) or mean ± standard deviation*.

*IW-21, INTERGROWTH-21^st^; Fenton, Fenton 2013. BMI, body mass index; EUGR, extrauterine growth restriction; RDS, respiratory distress syndrome; MV, mechanical ventilation; NEC, necrotizing enterocolitis; PDA, patent ductus arteriosus; ROP, retinopathy of prematurity; BPD, bronchopulmonary dysplasia; PVL, periventricular leukomalacia; HIV, intraventricular hemorrhage*.

* *True EUGR excludes IUGR (denominator IW-21 and Fenton are n = 412 and n = 402 respectively)*.

In dynamic true EUGR (decrease of more than 1 SD at discharge in non-IUGR patients), prevalence was 39.6% in IW-21 and 52.7% in Fenton. Patients with dynamic true EUGR using IW-21 have a higher risk of having comorbidities than when we used true static EUGR. In dynamic true EUGR, relative risks of presenting each complication are higher, and the relationship between the development of periventricular leukomalacia (PVL) and dynamic true EUGR is added.

Dynamic true EUGR according to IW-21 showed a history of significantly higher incidence of sex male, intubation during resuscitation at birth, RDS, mechanical ventilation, late sepsis, anemia requiring transfusion, NEC, PDA, hypotension, parenteral nutrition at 28 days, ROP ≥stage 2, BPD, PVL, and grade 3–4 IVH. In dynamic true EUGR according to Fenton, there was no association with male sex, hypotension, leukomalacia, and grade 3–4 IVH, but there was association with the rest of the morbidity levels.

Logistic regression analysis with the risk factors for the development of true EUGR (static and dynamic) is presented in Table 7. Independent variables related to the development of static “true EUGR” using IW-21 were GA, birth weight, male sex, RDS, anemia, ROP ≥stage 2, and NEC. Independent variables related with static “true EUGR” according to Fenton were GA, birth weight, male sex, RDS, and anemia. Independent variables related to the development of dynamic “true EUGR” using IW-21 were lower GA, male sex, RDS, and anemia. In dynamic “true EUGR” according to Fenton, independent variables related were male sex, RDS, and ROP ≥stage 2.

**Table 7:** Logistic regression analysis of risk factor for static and dynamic true EUGR with Fenton and IW-21.

	**B**	**Error standard**	***p* value**	**OR**	**95% CI**
**Static true EUGR for INTERGROWTH-21**^**st**^					
Gestational age (weeks)	0.944	0.162	<0.0001	2.57	1.87–3.52
Birth weight (g)	−0.008	0.001	<0.0001	0.992	0.989–0.994
Male sex	1.32	0.3	<0.0001	3.77	2.08–6.81
RDS	0.768	0.318	0.016	2.15	1.15–4.02
Anemia	1.34	0.322	<0.0001	3.83	2.04–7.2
ROP ≥ 2	0.951	0.473	0.045	2.58	1.02–6.54
NEC	2.62	1.15	0.023	13.85	1.43–133.96
**Static true EUGR for Fenton 2013**					
Gestational age (weeks)	1.16	0.16	<0.0001	3.21	2.33–4.43
Birth weight (g)	−0.01	0.001	<0.0001	0.99	0.987–0.993
Male sex	0.632	0.283	0.025	1.88	1.08–3.27
RDS	1.27	0.30	<0.0001	3.56	1.94–6.53
Anemia	0.87	0.328	0.008	2.38	1.25–4.53
ROP ≥ 2	0.201	0.501	0.688	1.22	0.45–3.26
NEC	1.68	1.12	0.134	5.37	0.59–48.36
**Dynamic true EUGR for INTERGROWTH-21**^**st**^					
Gestational age (weeks)	−0.45	0.13	0.001	0.63	0.49–0.828
Birth weight (g)	0.002	0.001	0.06	1.002	1–1.004
Male sex	0.983	0.271	<0.0001	2.67	1.57–4.54
RDS	0.848	0.304	0.005	2.33	1.28–4.23
Anemia	0.819	0.288	0.004	2.26	1.29–3.98
ROP ≥ 2	0.668	0.45	0.14	1.95	0.79–4.76
NEC	21.18	12,879.7	0.999	1.5E8	0.00–
**Dynamic true EUGR for Fenton 2013**					
Gestational age (weeks)	−0.23	0.12	0.059	0.791	0.62–1.009
Birth weight (g)	0.000	0.001	0.9	1	0.998–1.002
Male sex	0.518	0.259	0.045	1.67	1.01–2.78
RDS	0.79	0.27	0.003	2.22	1.3–3.79
Anemia	0.802	0.28	0.005	2.22	1.26–3.92
ROP ≥ 2	1.11	0.54	0.042	3.05	1.04–8.95
NEC	20.5	12,827.69	0.99	8.0E8	0.00–

*RDS, respiratory distress syndrome; NEC, necrotizing enterocolitis; ROP, retinopathy of prematurity*.

### Influence of the First 28 Days of life

**Figure 2:**
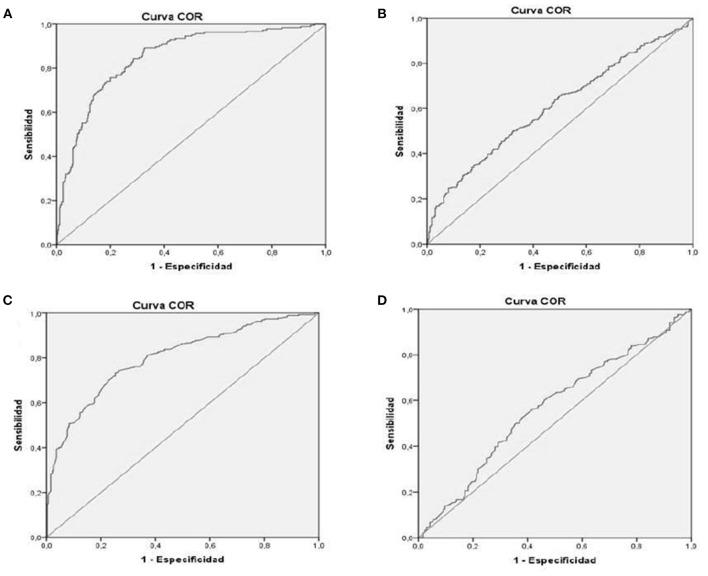
ROC cube: relationship between decrease Z score in the first 28 days of life and risk of EUGR **(A)** INTERGROWTH-21st dynamic-EUGR. **(B)** INTERGROWTH-21st static-EUGR. **(C)** Fenton dynamic-EUGR. **(D)** Fenton static-EUGR). EUGR, Extrauterine growth restriction.

Greater decrease in W z-score (IW-21 and Fenton) in the first 28 days of life was directly related with greater risk of static and dynamic EUGR at hospital discharge, more important with dynamic criteria and with IW-21 standards (Figure 2). In IW-21, ROC-AUC in dynamic EUGR was 0.849 (95% CI 0.816–0.882), and ROC-AUC in static EUGR was 0.610 (95% CI 0.566–0.654). In Fenton, ROC-AUC in dynamic EUGR was 0.805% (95% CI 0.764–0.835), and ROC-AUC in static EUGR was 0.566 (95% CI 0.514–0.617).

### Changes Over Time

Dividing the period into octens (2002–2009 vs. 2010–2017), we observed that there were no significant differences in the prevalence of newborns <28 weeks (21.5 vs. 17%); neither in IUGR (Fenton 34.2 vs. 39%, IW-21 32.7 vs. 37.7%) nor in static EUGR (Fenton 75.2 vs. 72.4%, IW-21 56.7 vs. 51.1%).

However, we found a significantly lower prevalence of dynamic EUGR (Fenton 51.5 vs. 36.5%, IW-21 37.1 vs. 21%) (*p* < 0.0001).

## Discussion

In this retrospective study, we analyzed the prevalence of IUGR at birth and EUGR at discharge in VLBW infants using two different growth charts (IW-21 and Fenton 2013) and in EUGR using three different criteria. The use of these different classifications is important because of the medium- and long-term consequences that they can define. In our series, with the use of both graphs and a cutoff point in the 10th percentile, IUGR frequency was 33.2, 25.4, and 30.4% for W, L, and HC, respectively.

When we analyzed IUGR data independently with each growth chart, we observed that IW-21 and Fenton classify them similarly (IW-21: 35.1, 28.8, and 34.8% vs. Fenton: 36.5, 26.8, and 34.8%, for W, L, and HC, respectively) with a high level of agreement (Kappa > 0.8). When we compared the morbidity between IUGR for both vs. non-IUGR for both charts, we observed that IUGRs for both had less frequency of morbidities, probably related to having a higher GA (32.3 ± 2.2 vs. 29.2 ± 2.2 weeks, *p* < 0.000). No significant differences were observed in morbidities of patients in whom both graphs differ when defining IUGR. IUGR frequency in our series is higher than that of other studies (10–20%) (15, 20, 21), probably due in large part to the use of birth weight instead of GA as a selection criterion. This frequency is similar to the total of the SEN1500 network (33.3%) (22).

Lebrao et al. (23) in a retrospective study with 26–33 weeks preterms (*n* = 173) showed that IW-21 and Fenton were similar for classifying IUGR by weight (35.2 vs. 39.2%). In Barreto's series, which included 2,489 newborns between 34 and 41 weeks, fewer patients were only identified as IUGR using IW-21 (13 vs. 8.7%) (21). However, Tuzun et al. (17), using newborns under 32 weeks GA, identified more IUGR (15 vs. 12%) with IW-21.

Today, there is great controversy about which classification to use to define EUGR in VLBW newborns and which one best defines their long-term prognosis. Classically, static definition has been used to define EUGR, which includes all patients below the 10th percentile at hospital discharge after neonatal admission. Recently, there has been talk of dynamic EUGR, including patients who, from birth to discharge, experienced a decrease in weight >1 SD. In addition, in order to isolate the confounding effect of IUGR, whose postnatal growth may be influenced by prenatal factors, the concept of true EUGR was introduced, defining patients EUGR without history of IUGR.

When we analyzed the frequency of static EUGR (less than the 10th percentile at discharge), using the IW-21 and Fenton charts, we observed a high disparity in weight but a good match in length and HC (IW-21: 53.9, 57.6, and 25.9% vs. Fenton: 73.8, 63.6, and 23.5% W, L, and HC, respectively). These data are influenced by IUGR frequency, so that in our case, only 3 and 12% of them (Fenton and IW-21, respectively) reached a W 10th percentile at discharge.

Dynamic analysis of postnatal growth reflects better how this has been, isolating the positive effect of IUGRs and the negative effect of non-IUGRs. Thus, 69.3 and 89.2% of the IUGR, according to Fenton and IW-21, respectively, reached a weight >1 SD at discharge. Overall, 29.0, 41.4, and 9.8% of IUGRs had dynamic EUGRs for W, L, and HC, respectively, for both charts. The disparity between both charts was lower than with the static method (IW-21: 29.3, 43.8, and 12.6% vs. Fenton: 44.3, 58.5, and 13.7% for W, L, and HC, respectively), with IW-21 being more restrictive for all three measures. The percentage of misclassified cases was lower with IW-21 than with Fenton for all three: 0.3, 2.4, and 2.8% vs. 15.3, 17.1, and 3.9%, IW-21 vs. Fenton for W, L, and HC, respectively.

Ávila-Álvarez (24) used the Fenton curves in a cohort of 130 VLBW and obtained an 59.2% EUGR prevalence. Figueras-Aloy (16) obtained 50% EUGR prevalence (W below the 10th percentile in weeks 34–36) using IW-21 references in a cohort of 479 children under 32 weeks born between 2003 and 2014. In our series, with the use of these criteria, it would be 73.8% (Fenton) and 53.9% (IW-21) due to the high proportion of IUGR patients in our series.

If we focus on <1,000 g birth weight infants (*N* = 161), only 23.6% achieved a discharge W above the 10th percentile using IW-21 and 13% using Fenton. These results are very similar to those that had already been reported in another publication (25). We observed a good concordance between both classifications in this group (Kappa > 0.6).

When we exclude IUGR, static and dynamic true EUGR prevalence for W was, respectively, 35.7 and 39.6% for IW-21 and 60.4 and 52.7% for Fenton. The percentage of misclassified cases as non-EUGR by IW-21 was much lower than that by Fenton (1 vs. 98%). The agreement of both graphs for L and HC was much better, especially for the static calculation. In any case, our data are lower than those of Figueras-Aloy (16), who obtained a true EUGR prevalence of 42.7% by IW-21, and higher than those of Tuzun et al. (17), who published 24 and 37.7% for IW-21 and Fenton, respectively.

Patients who experienced true EUGR have had more frequent perinatal morbidity during their admission (late-onset sepsis, RDS, anemia requiring transfusion, hypotension, BPD, ROP ≥ stage 2, PDA, parenteral nutrition at 28 days of life, and NEC) and longer neonatal admission. A greater relative risk is observed for having had these pathologies if we use dynamic criteria vs. static criteria to define EUGR, and in both cases greater statistical association using IW-21. Therefore, dynamic EUGR defined with IW-21 references is associated with greater complications in neonatal period and may better represent EUGR.

Among independent risk factors for the development of true EUGR, we found, as Figueras-Aloy did, male sex, birth weight, GA, and RDS (16). In our series, BPD was not found to be an independent risk factor.

In our study, greater decrease in the z-score in W (IW-21 and Fenton) in the first 28 days of life was directly related to a greater risk of static and dynamic EUGR at hospital discharge, but with greater power of relation with dynamic criteria and with IW-21 standards. This implies that the first weeks of life of VLBW represent a critical period of growth, with a high probability of morbidity and difficulties in growth, and the repercussion on growth will continue at hospital discharge. Prioritizing and emphasizing nutrition in the first 28 days of life are essential.

According to the criteria and chart we use, EUGR prevalence in VLBW varies substantially within the same series: from 73.8% of patients using static criteria in the Fenton graphs to 29.3% using dynamic criteria with IW-21 standards. Therefore, up to twice as many patients can be diagnosed with EUGR according to the criteria we use; hence, the importance of determining which classification is better.

In our series, IW-21 seems stricter than Fenton for classifying EUGR (static, dynamic, and true EUGR). Furthermore, patients diagnosed by IW-21 as EUGR for W had more frequent neonatal morbidity during their admission.

Kim et al. (26) obtained a similar result after comparing the dynamic EUGR and static EUGR in both graphs in a cohort of 1,356 preterm infants with GA <28 weeks. Tuzun et al. (17) also used static criteria to define EUGR and made a comparison between the Fenton and IW-21 curves, including 248 children under 32 weeks with an IUGR percentage of 12%, observing a lower prevalence of EUGR using IW-21 (31.5 vs. 40%). Reddy et al. (15) also obtained a lower incidence of EUGR with IW-21 compared with Fenton (48 vs. 55%), after analyzing 603 under 32 weeks with a proportion of IUGR of 15%. In a European multicountry cohort, EUGR (discharge weight <10th percentile) using the Fenton charts varied from 24% (Sweden) to 60% (Portugal) and using IW-21 from 13% (Sweden) to 43% (Portugal) (27). A wide variation in rate of EUGR is related to heterogeneity of inclusion criteria and used definition in different reports.

This study and other previous ones highlight the need to standardize criteria and the evaluation method of EUGR, which allow to compare results and to generate hypotheses to improve nutrition in neonatal units and to perform studies on its long-term implication.

Several studies link long-term EUGR with growth retardation and adverse neurodevelopmental, although this last association is not clearly demonstrated (28). Although morbidity associated with prematurity contributes to EUGR, nutrition is the most significant determinant. Adequate nutritional support during the hospital stay is critical to reduce EUGR rate. However, if it is excessive, this can lead to an increase in fat mass and a higher future risk of non-communicable diseases such as obesity or metabolic syndrome (29).

Although more studies are necessary, we believe that IW-21 could be the best standard for assessing postnatal growth, in terms of both the method of construction and its correlation with EUGR risk factors. We also consider that the change in z-score reflects postnatal growth better than static analysis at discharge.

Among the limitations of our study, we should note the long-term recruiting time in order to include as much low VLBW infants as possible, having in count the low VLBW incidence we have in our unit. In this period of time, there were changes in neonatal management according to the latest recommendations. However, this study analyzes a very large sample of VLBW infants to evaluate IUGR and EUGR prevalence according to classic curves (Fenton) and new ones, although little used in daily practice (IW-21), including a comparison between different ways of defining EUGR (static and dynamic) and associated factors.

## Conclusions

Concordance between the Fenton and IW-21 graphs for IUGR is good, but there is less agreement in EUGR, with IW-21 being more restrictive. However, patients diagnosed by IW-21 as EUGR are more likely to have had neonatal morbidities, especially if we use the dynamic definition of EUGR. Greater decrease in z-score in W in first 28 days predicts EUGR (all types) risk at discharge in both growth charts. In our study, we cannot conclude that one graph is better than the other.

## Data Availability Statement

The raw data supporting the conclusions of this article will be made available by the authors, without undue reservation.

## Ethics Statement

The studies involving human participants were reviewed and approved by Research Ethics Committee of Principado de Asturias (CEIm PA, SPAIN). Written informed consent to participate in this study was provided by the participants' legal guardian/next of kin.

## Author Contributions

GS-S, LG-G, and EG-L contributed to conception and design of the study. BF-C organized the database. LM-F and GS-S performed the statistical analysis. LG-G wrote the first draft of the manuscript. SL-V, MS-R, and RA-L wrote sections of the manuscript. All authors contributed to manuscript revision, read, and approved the submitted version.

## Conflict of Interest

The authors declare that the research was conducted in the absence of any commercial or financial relationships that could be construed as a potential conflict of interest.

## Abbreviations

AAP: American Academy of Pediatrics
BMI: body mass index
BPD: bronchopulmonary dysplasia
CRIB: Clinical Risk Index for Babies
EUGR: extrauterine growth restriction
GA: gestational age
HC: head circumference
IUGR: intrauterine growth restriction
IVH: intraventricular hemorrhage
IW-21: INTERGROWTH-21st
L: length
MV: mechanical ventilation
NEC: necrotizing enterocolitis
non-IUGR: non-intrauterine growth restriction
PDA: patent ductus arteriosus
PMA: postmenstrual age
PVL: periventricular leukomalacia
RDS: respiratory distress syndrome
ROP: retinopathy of prematurity
SD: standard deviation
VLBW: very low birth weight
W: weight
WHO: World Health Organization.
